# Extramacular Retinal Hole Following Intravitreal Dexamethasone Implant: Case Report

**DOI:** 10.4274/tjo.galenos.2019.98975

**Published:** 2019-06-27

**Authors:** Cansu Ekinci, Alp Kayıran, Hakan Özdemir

**Affiliations:** 1 Bezmialem Vakıf University Faculty of Medicine, Department of Ophthalmology, İstanbul, Turkey; 2 Yeditepe University Ophthalmology Research and Application Center, Department of Ophthalmology, İstanbul, Turkey

**Keywords:** Intravitreal dexamethasone implant, Ozurdex, retinal hole

## Abstract

The intravitreal dexamethasone implant Ozurdex is indicated for the treatment of macular edema due to diabetes and branch retinal vein occlusion. While the most common ocular side effects are elevated intraocular pressure and cataract formation, rare complications related to the injection have been reported. We present a case with extramacular retinal hole after Ozurdex injection.

## Introduction

The intravitreal dexamethasone implant (Ozurdex^®^, Allergan Inc., Irvine, CA, USA), which provides sustained drug release when injected into the vitreous cavity, is used in the treatment of macular edema due to branch retinal vein occlusion (BRVO) as well as many other retinal diseases.^1^ Although intraocular pressure elevation and cataract are the most common complications after Ozurdex injection, there have also been reports of retinal tears, retinal hemorrhage, intralenticular implantation, subretinal injection, implant migration to the anterior chamber, endophthalmitis, and macular hole.^2^ In this case report, we present a patient with extramacular retinal hole, a rarely reported complication after Ozurdex injection.

## Case Report

**Figure 1:**
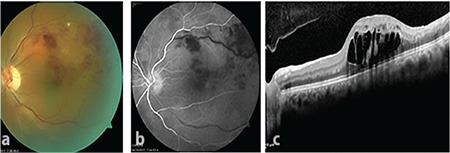
At presentation, a) color fundus image showed superotemporal branch retinal vein occlusion; b) fundus fluorescein angiography showed late filling and dilation of the superotemporal vein and areas of capillary nonperfusion; and c) optical coherence tomography showed macular thickening and cystoid edema

A 54-year-old man presented with complaints of decreased visual acuity in his left eye for approximately 1 week. His medical history included no systemic disease other than hypertension that had been present for 5 years and was controlled with medical treatment. In ophthalmologic examination, his corrected visual acuity was 1.0 in the right eye and 0.2 in the left eye. He had no history of previous ocular surgery, and anterior segment examination was normal. Fundus examination revealed no pathology in the right eye but BRVO was detected in the superotemporal region of the left eye (Figure 1a). Intraocular pressure was 15 mmHg in the right and 14 mmHg in the left eye. Fundus fluorescein angiography of the left eye showed late filling, dilation, and increased tortuosity of the superotemporal retina vein and areas of capillary nonperfusion consistent with BRVO (Figure 1b). Spectral domain optical coherence tomography (OCT) demonstrated retinal thickening (710 µm) and cystoid macular edema (Figure 1c). The patient was diagnosed with macular edema associated with BRVO and Ozurdex was injected. The injection was done in aseptic conditions from the superotemporal quadrant 4 mm from the limbus using the recommended standard procedure. During implantation, slight deflation of the globe and momentary hypotony were observed immediately after inserting the sharp tip of the implant through the sclera and before pulling the trigger, despite the absence of vitreous leakage. Vitreous leakage or hypotony were not observed after injection and no complications were noted in routine follow-up examination the next day.

**Figure 2:**
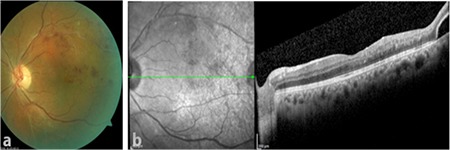
At follow-up 1 month after Ozurdex injection, a) color fundus image showed regression of superotemporal hemorrhages and b) optical coherence tomography showed resolution of cystoid macular edema and normalization of the foveal contour

**Figure 3:**
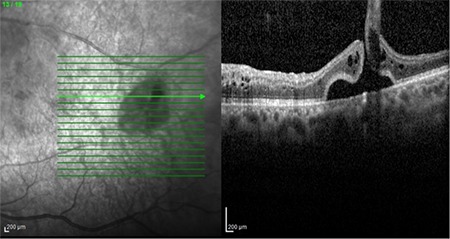
Optical coherence tomography section passing through the hole temporal of the macula at 1 month after injection

At 1-month follow-up, the patient’s visual acuity had increased to 0.5. Hemorrhage was observed in the superotemporal region on fundus examination (Figure 2a). Macular OCT examination revealed that the cystoid macular edema had resolved, foveal thickness was 266 µm, and foveal contour had normalized (Figure 2b). A full-thickness retinal hole about 1 disc diameter in size surrounded by sporadic hemorrhages was noted in the temporal region of the macula (Figure 3). The patient was informed of their condition and laser photocoagulation was performed on the ischemic areas and around the retinal hole. At follow-up 4 months after injection, visual acuity in the left eye was 0.3 and intraocular pressure measured by Goldmann applanation tonometry was 15 mmHg in both eyes. Central macular thickness had increased to 613 µm. The patient was given a second Ozurdex implant. He was last seen 1 month after the second injection. At that time, his visual acuity increased to 0.4 in the left eye. The retina was attached and the laser spots showed pigmentation. Central macular thickness had decreased to 284 µm.

## Discussion

Ozurdex is an intravitreal sustained-release dexamethasone implant known to be effective in the treatment of macular edema due to BRVO. The most commonly observed side effects are increased intraocular pressure and cataract formation, though other complications have been associated with the implant, such as migration into the anterior chamber, intralenticular implantation, confinement to Berger’s space, and conditions like endophthalmitis, vitreomacular traction, and macular hole.^2^

Extramacular retinal hole following Ozurdex injection, as seen in our case, has only been reported previously by Christensen et al.^3^ Their case report was based on the patient’s anamnesis, which suggested that the eccentric macular hole that developed after receiving an Ozurdex injection abroad was likely due to direct contact of the Ozurdex implant with the retina. The patient did not have records from before the Ozurdex injection. In an experimental apparatus created for this case report, the authors determined that the force created by the implant at a distance of 16 mm with Ozurdex applicator was 0.77 Newton (N) in air and 0.024 N in BSS. They reported that these values were lower than the 0.1-0.2 N necessary for a foreign body to damage the retina according to previous studies. In this case, which they referred to as the “magic bullet”, the authors believed that no mechanism other than direct contact by the implant could have created the retinal hole and suggested that this complication may be attributable to the retina becoming more susceptible to trauma in chronic retinal disease.

One point to consider here is the relationship between the speed at which the trigger is pushed and the velocity with which the implant is released. Meyer et al.^4^ reported in an experimental study that the Ozurdex implant exited the applicator at a speed of 0.8 m/s and decelerated progressively, and that its deceleration was more rapid in the vitreous compared to water. They concluded that the retinal impact energy calculated in their analyses did not reach the previously reported levels necessary to reach the retina. In addition, the authors followed the patient without treatment and reported that the hole was stable. For our patient, however, we preferred to treat with laser photocoagulation because the hole appeared to be large and causing traction.

In our case, the momentary hypotony observed immediately before pushing the trigger during injection may have shortened the distance between the entry site and retina, thus allowing the implant to cause direct damage to the retina. Therefore, we believe that patients who exhibit globe softening during implantation require special care, and that at the very least, the clinician should attempt to aim the implant toward the extramacular area.
